# Effects of Unilateral Stimulation in Parkinson's Disease: A Randomized Double-Blind Crossover Trial

**DOI:** 10.3389/fneur.2021.812455

**Published:** 2022-01-20

**Authors:** Zhitong Zeng, Linbin Wang, Weikun Shi, Lu Xu, Zhengyu Lin, Xinmeng Xu, Peng Huang, Yixin Pan, Zhonglue Chen, Yun Ling, Kang Ren, Chencheng Zhang, Bomin Sun, Dianyou Li

**Affiliations:** ^1^Department of Neurosurgery, Center for Functional Neurosurgery, Ruijin Hospital, Shanghai Jiao Tong University School of Medicine, Shanghai, China; ^2^Institute of Science and Technology for Brain-Inspired Intelligence, Fudan University, Shanghai, China; ^3^Gyenno Science Co., LTD., Shenzhen, China

**Keywords:** deep brain stimulation (DBS), Parkinson's disease, globus pallidus interna, subthalamic nucleus, personalized treatment

## Abstract

**Introduction:**

Previous studies have shown that subthalamic nucleus (STN) and unilateral globus pallidus interna (GPi) are similarly effective in the deep brain stimulation (DBS) treatment of motor symptoms. However, the counterintuitively more common clinical application of STN DBS makes us hypothesize that STN is superior to GPi in the treatment of motor symptoms.

**Methods:**

In this prospective, double-blind, randomized crossover study, idiopathic PD patients treated with combined unilateral STN and contralateral GPi DBS (STN in one brain hemisphere and GPi in the other) for 2 to 3 years were enrolled. The MDS UPDRS-III total score and subscale scores for axial and bilateral limb symptoms were assessed preoperatively and at 2- to 3-year follow-up in four randomized, double-blinded conditions: (1) Med–STN+GPi–, (2) Med–STN–GPi+, (3) Med+STN+GPi–, and (4) Med+STN–GPi+.

**Results:**

Eight patients had completed 30 trials of assessment. Compared with the preoperative Med– state, in the Med–STN+GPi– condition, the cardinal symptoms in both sides of the body were all improved. In the Med–STN–GPi+ condition, symptoms of the GPi-stim limb were improved, while only tremor was improved on the ipsilateral side, although all axial symptoms showed aggravation. Compared with the preoperative Med+ state, in the Med+STN+GPi– state, cardinal symptoms were improved on both sides, except that tremor was worsened on the STN-stim side. In the Med+STN–GPi+ state, the overall motor symptoms were aggravated compared with the preoperative Med+ state. Most axial symptoms worsened at acute unilateral STN or GPi DBS onset, compared to both preoperative Med– and Med+ states. No side effects associated with this study were seen.

**Conclusions:**

Improvement in motor symptoms was greater in all sub-scores favoring STN. The effects of STN+ were seen on both sides of the body, while GPi+ mainly acted on the contralateral side.

## Introduction

Deep brain stimulation (DBS) is a well-established surgical intervention for patients with advanced Parkinson's disease (PD), especially those with medication-resistant motor symptoms, motor fluctuations, or levodopa-induced dyskinesia (1, 2). However, choosing a suitable stimulation target to maximize clinical outcomes while minimizing side effects remains a challenge.

The subthalamic nucleus (STN) and globus pallidus interna (GPi) are the two main targets in large randomized controlled trials in which patients with comparable clinical and demographic characteristics are randomized to receive either GPi DBS or STN DBS. Studies have demonstrated similar effects for both targets on motor symptom improvement (3). Unfortunately, for highly heterogeneous diseases, such as PD, these randomized controlled trials, designed to be conducted among different patients yielded inconsistent results, even when sufficient numbers of patients were included.

Most studies have investigated the differences between STN and GPi DBS either unilaterally or bilaterally in different patients and presented evidence for similar effectiveness of STN and GPi on motor symptoms (4). However, significantly more STN DBS were performed clinically, which made us wonder whether STN is more trusted than GPi with respect to its treatment effect. Therefore, we hypothesized that STN is superior to GPi in the treatment of motor symptoms.

In this study, we aimed to elucidate the nuances between STN and GPi DBS in PD patients. We conducted an intra-patient comparison by investigating the acute turning-on effects of unilateral STN stimulation vs. unilateral GPi stimulation on motor symptoms within each patient who had received a treatment comprising combined unilateral STN and contralateral GPi DBS. The asymmetrically targeted DBS treatment was first applied in our previous study to address the assumption of different therapeutic effects with unilateral STN and contralateral GPi DBS. Our previous research (5) showed that at the 1-year follow-up, this approach represented an effective and well-tolerated DBS treatment option for selected patients with advanced PD, incurring no significant increase in side effects.

## Methods

### Standard Protocol Approvals, Registrations, and Patient Consents

This study was conducted under the supervision of the ethical committee in Ruijin Hospital, Shanghai Jiao Tong University School of Medicine. All patient's consent was collected according to the Declaration of Helsinki. This study is registered on clinicaltrial.gov (clinicaltrial.gov NCT04255719).

### Trial Design

**Figure 1:**
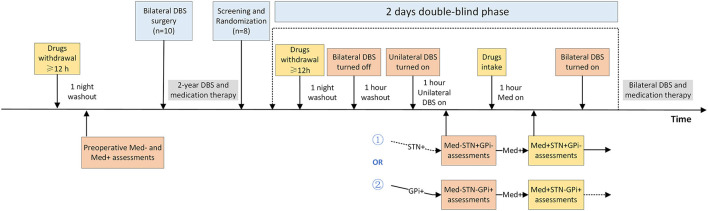
Randomized, double-blind, crossover design of the study. This study involved two time periods, before and 2 to 3 years after surgery. Some patients (*n* = 10) were assessed for motor symptoms in Med– and Med+ states preoperatively and underwent asymmetric target (unilateral STN, contralateral GPi) DBS treatment. After parameter optimization, the patients were treated with medication and bilateral DBS. We recruited and screened patients 2 to 3 years after surgery (*n* = 8) and randomized them into two groups. One group of patients was evaluated first in the STN+GPi– state and then the next day in the STN–GPi+ state; the other group was the opposite, STN–GPi+ then STN+GPi–. Patients stopped intaking antiparkinsonian drugs the night before the assessment, ensuring that they were off medication for more than 12 h. Bilateral DBS was turned off and washed out for 1 h before assessment. Afterwards, the unilateral DBS was turned on and motor symptoms were assessed in the Med– condition. Following the Med– condition assessment, the patient took the medication and was assessed 1 h later in the Med+ condition. After completion of the first day's assessment, the patient's bilateral DBS was turned on, although medication withdrawal continued for more than 12 h. Assessment of the contralateral DBS on state was performed on the second day. Patients were blinded for all assessment procedures and resumed prior bilateral DBS and medication treatment after the four-state assessments. DBS, deep brain stimulation; GPi, globus pallidus interna; STN, subthalamic nucleus.

This was a prospective double-blind randomized crossover study designed to compare the acute effect of unilateral STN and GPi stimulation on motor symptoms in several patients with PD. Participants with advanced PD who had previously undergone combined unilateral STN and contralateral GPi DBS were screened based on the inclusion and exclusion criteria. Following recruitment, participants were comprehensively evaluated under four randomized, double-blind conditions: (1) Med–STN+GPi–, (2) Med–STN–GPi+, (3) Med+STN+GPi–, and (4) Med+STN–GPi+. The symbol + means on, while – means off. The **intervention** section explains the details of these conditions. All participants and trained assessors were blinded to the conditions, and patients were randomly assessed over the course of two continuous days (Figure 1).

### Patients

Participants were recruited from the Department of Functional Neurosurgery at Ruijin Hospital, Shanghai Jiao Tong University School of Medicine (Shanghai, China). A total of 10 patients with PD underwent combined unilateral STN and contralateral GPi DBS from September 2017 to September 2018. Following recruitment in April 2020 and screening, eight patients who had received the surgery for 2 to 3 years were included in this study. Supplementary Material 1 explains the surgical procedure. The inclusion criteria were: (1) diagnosis of idiopathic PD; (2) age between 55 and 75 years, both male and female; (3) treatment with combined unilateral STN and contralateral GPi DBS for 2 to 3 years with optimal parameters for 3 months; and (4) a Hoehn-Yahr (H-Y) stage of less than 4 in the medication-off state. The exclusion criteria were: (1) history of serious psychosis; (2) history of intractable epilepsy (i.e., seizures); (3) diagnosis of severe cardiac, liver or kidney diseases, or other serious health conditions; (4) dementia (A Mini-Mental State Examination score of <24), inability to comprehend the experimental protocol or voluntarily provide informed consent; (5) lack of cooperation; (6) poorly controlled depression or anxiety. The patients in this study overlapped partly with those in our previous study published in 2020; (5) those were patients 3, 7 and 8. Additionally, according to the asymmetry index, patients were divided into a symmetric group (asymmetry index <0.15, both Med– and Med+ conditions before surgery) and an asymmetric group (asymmetry index ≥ 0.15, either Med– or Med+ conditions before surgery), and the corresponding subgroup analysis was performed. The asymmetry index was a left-extremity to right-extremity ratio in the MDS UPDRS-III, which was calculated using the formula (left extremity – right extremity) / (left extremity + right extremity) (6, 7). A team of experienced multidisciplinary DBS specialists made the clinical decision regarding the specific DBS target to be used in each patient. That was, unilateral STN DBS was applied to treat the more severe side since we hypothesized that STN is more effective than GPi. We highlighted three cases here. Patient 4 underwent DBS surgery because of the adverse effect of postural hypotension after taking the medication. Patient 7 had opposite asymmetry indices in the Med+ and Med– states, so STN was applied to the left hemisphere due to higher severity of the right limb in the Med– state (3). Patient 3 had the same scores on bilateral limbs in the Med+ and Med– states, and we applied unilateral STN DBS to the left hemisphere because there is evidence of a left-hemispheric dominance for appendicular movements and a right-hemispheric dominance for axial motor control (8, 9).

### Interventions

#### Unilateral DBS of STN

Bilateral stimulation was turned off for an hour (10), and unilateral STN DBS was turned on afterwards. Participants were asked to complete a comprehensive set of assessments under unilateral STN stimulation in the Med– state. Participants were further required to complete the second set of assessments in the Med+ state 1 h after taking regular medications,.

#### Unilateral DBS of GPi

Unilateral GPi DBS was delivered after bilateral stimulation was turned off for an hour. The study protocol was identical to that used in the unilateral STN DBS intervention but was performed on a different day. After all these assessments, bilateral DBS will be turned on again and returned to normal treatment status.

#### Concomitant Interventions

Participants were asked to stop taking antiparkinsonian drugs for 12 h to stay in the Med– state until they completed the first set of assessments. Regular medication was taken 1 h before the second set of assessments to maintain a Med+ state. All processes were repeated for the contralateral target on the next day.

### Randomization and Blinding

The testing sequence of the treatment conditions was randomly assigned in a counterbalanced manner on the scheduled days. The order of the DBS conditions was determined by the clinician who randomly picked up one of the eight folded sheets with different conditions written on them (half of the first day GPi; half of the first day STN) but was not allowed to participate in any rating or evaluation. Throughout the study, all participants, raters, and statisticians were blinded to treatment conditions. A movement disorder specialist was responsible for programming. In addition, motor symptom evaluation in this experiment was performed by an experimenter who was blinded to the study protocol and did not participate in data analysis or interpretation. Two raters who were blinded to the conditions conducted the video assessments independently, after which the average rating scores were calculated. For subscores with large deviancy, the final scores were determined after re-evaluation.

### Trial Outcomes

Acute turning-on effects of unilateral STN stimulation vs. unilateral GPi stimulation on motor symptoms in each patient were compared as the primary outcome. Motor symptoms were defined by the MDS UPDRS-III scores which ranged from 0 to 132, with higher scores indicating more severe motor symptoms (11). To gain insight into the specific effects of each target, we classified the MDS UPDRS-III subscales into three categories: (1) axial signs, as measured by scores on speech, facial expression, arising from a chair, posture, gait, freezing of gait, and posture stability; scores could range from 0 (no axial signs) to 28 (severe axial signs); (2) STN-stimulated contralateral limb symptoms; and (3) GPi-stimulated contralateral limb symptoms. Limb symptom severity was measured using the subscale scores of the corresponding limb on rigidity, finger tapping, hand movements, hand pronation supination, toe-tapping, leg agility, posture tremor, kinetic tremor, and resting tremor amplitude; scores could range from 0 (no limb symptoms) to 52 (severe limb symptoms). The Berg Balance Scale (BBS) was also compared as a second outcome at the 2- to 3-year follow-up. The patient's daily dose of antiparkinsonian medication was converted into a levodopa equivalent daily dose (LEDD).

### Data Analysis

There were two types of comparisons conducted in this study. The first was the comparison between unilateral STN+ and GPi+ within the same patient group in Med– and Med+ conditions, and the second was the comparison between asymmetric and symmetric groups for the different patient groups in the same condition. Before the comparisons, the Shapiro-Wilk test was used to test the normality of data in each group, yielding the W statistic and *P*-value reflecting the evaluation criteria of distribution. For normally distributed data, a parametric test of the Student's *t*-test was used to assess the difference between groups. For the non-normally distributed data, the non-parametric Wilcoxon test was applied to compare the differences. The first comparison mentioned was based on the paired Student's *t*-test and Wilcoxon signed-rank test. The second comparison was based on the independent Student's *t*-test and Wilcoxon rank-sum test. All three tests mentioned were two-tailed tests with a *P*-value <0.05 reflective of statistical significance. Bonferroni correction was applied for adjustment of multiple testing. Statistical calculations and techniques were performed using R-4.0.2.

### Data Availability

Original data is available upon reasonable request.

## Results

### Patients

Eight patients completed 30 trials of assessment at 2 to 3 years after DBS operation, of which 16 met the Med–STN+GPi–/ Med–STN–GPi+ conditions and 14 met the Med+STN+GPi–/ Med+STN–GPi+ conditions. The main demographics and clinical characteristics of the patients are presented in Table 1. All the actual postoperative lead locations were in accordance with the preoperative plan. The stereotactic coordinates and programming parameters of each patient are shown in Supplementary Table 1.

**Table 1:** Demographic and clinical characteristics of each patient.

**Patients**	**Sex**	**Age at surgery (yrs)**	**Disease duration at surgery (yrs)**	**LEDD at surgery (mg)**	Asymmetry index^*^ **Med–, Med+**	**Group**	**Target**	**Follow-up Period (months)**
Patient 1	Male	74	7	700	0.09	Symmetric group	R-GPi	32
					0.03		L-STN	
Patient 2	Female	61	26	525	0.00	Symmetric group	R-STN	23
					0.00		L-GPi	
Patient 3	Female	69	9	500	0.04	Symmetric group	R-GPi	31
					0.02		L-STN	
Patient 4	Male	64	8	150	0.10	Symmetric group	R-GPi	32
					0.14		L-STN	
Patient 5	Male	73	4	425	−0.21	Asymmetric group	R-STN	29
					−0.10		L-GPi	
Patient 6	Female	58	4	787.5	−0.67	Asymmetric group	R-STN	32
					−0.50		L-GPi	
Patient 7	Male	72	18	1,050	0.15	Asymmetric group	R-GPi	32
					−0.20		L-STN	
Patient 8	Male	58	5	798.25	0.29	Asymmetric group	R-GPi	36
					0.38		L-STN	

*LEDD, levodopa equivalent daily dose; R-GPi, right unilateral stimulation of the globus pallidus interna; L-STN, left unilateral stimulation of the subthalamic nucleus; R-STN, right unilateral stimulation of the subthalamic nucleus; L-GPi, left unilateral stimulation of the globus pallidus interna. Asymmetric group, patients with asymmetry index ≥ 0.15 at either Med– or Med+ conditions before surgery; Symmetric group, patients with asymmetry index <0.15 at both Med– and Med+ conditions before surgery. Mean age at surgery, 66.1 ± 6.3 yrs; mean disease duration at surgery, 10.1 ± 7.3 yrs; mean LEDD at surgery, 617.0 ± 276.0 mg*.

* *The asymmetry index was calculated as the absolute difference between the total of the items for each side divided by the sum of the items for both sides [(left extremity – right extremity)/ (left extremity + right extremity)]. A higher asymmetry index indicated higher asymmetry in symptom severity or symptom types*.

### Acute Effects of Unilateral STN+/Med– vs. GPi+/Med–

We first analyzed the difference in treatment outcomes between unilateral STN+ and GPi+ in the Med**–** state compared to the preoperative Med– state. The mean total MDS UPDRS-III score was reduced by 26% in STN+/Med**–** but showed almost no change in GPi+/Med**–**. STN+ improved motor symptoms on both sides of the body, while GPi+ mainly on the GPi-stim side. Axial symptoms worsened in both STN+/Med**–** and GPi+/Med**–** states, but the deterioration was more pronounced in the GPi+/Med**–** state, especially with differences in symptoms of postural stability, posture, and global spontaneity of movement (Table 2).

**Table 2:** Motor symptoms in Med–STN+GPi– and Med–STN–GPi+ conditions before and 2 to 3 years after surgery (*n* = 8).

		**Baseline Med–^a^**	**Follow-up Med–**		**Percentage of change**		**Adjusted** ***P*****-value**		
			**STN+GPi–^b^**	**STN–GPi+**	**STN+GPi–**	**STN–GPi+**	**STNa**	**Gpi a**	**b**
**Total UPDRS-III**		54.12 ± 24.7	40.25 ± 16.54	53 ± 17.76^b^	−25.6%	−2.1%	0.3498	1	0.0279
Tremor		10.38 ± 7.31	4.75 ± 4.68	7 ± 5.45	−54.2%	−32.6%	0.2346	0.621	1
Rigidity		11.75 ± 4.43	8.5 ± 3.78	12.12 ± 4.05^b^	−27.7%	3.1%	0.5202	1	0.0936
Bradykinesia		21.38 ± 8.68	13.75 ± 7.61^a^	18.62 ± 9.24^b^	−35.7%	−12.9%	0.0234	1	0.0459
**STN-stim limb**	Tremor	5 ± 2.93	1.88 ± 1.89^a^	3.5 ± 2.93	−62.4%	−30.0%	0.1215	0.6099	0.7629
	Rigidity	5.12 ± 1.81	2.5 ± 1.77^a^	5.12 ± 2.3^b^	−51.2%	0	0.0753	1	0.0753
	Bradykinesia	11.75 ± 3.37	6.38 ± 3.62^a^	11.38 ± 5.37^b^	−45.7%	−3.1%	0.0018	1	0.0141
**GPi-stim limb**	Tremor	2.88 ± 2.8	2 ± 2.14	2 ± 1.85	−30.6%	−30.6%	1	0.9051	1
	Rigidity	4.38 ± 1.69	3.38 ± 1.92	3.75 ± 1.67	−22.8%	−14.4%	0.7572	1	1
	Bradykinesia	9.62 ± 5.42	7.38 ± 4.47	7.25 ± 4.59	−23.3%	−24.6%	0.4929	0.5775	1
**Axial signs**	Total axial score	10.62 ± 7.25	13.25 ± 5.31	15.25 ± 5.12^b^	24.8%	43.6%	0.513	0.2421	0.1239
	Speech	1.12 ± 1.13	1.38 ± 0.74	1.75 ± 0.89	23.2%	56.3%	1	0.6558	0.4467
	Facial expression	1.88 ± 1.13	2.12 ± 0.35	2 ± 0.93	12.8%	6.4%	1	1	1
	Arising from chair	1.25 ± 1.49	0.88 ± 0.99	1.38 ± 1.19	−29.6%	10.4%	1	1	0.2157
	Gait	1.62 ± 1.19	1.62 ± 0.52	1.75 ± 0.46	0.0%	8.0%	1	1	1
	Freezing of gait	0 ± 0	0.62 ± 0.74	0.75 ± 0.89	∞	∞	0.267	0.2841	1
	Postural stability	1.25 ± 1.49	2.25 ± 1.75	2.38 ± 1.06^a^	80.0%	90.4%	0.267	0.1383	1
	Posture	2 ± 1.07	2.12 ± 0.83	2.5 ± 1.07^a^	6.0%	25.0%	1	0.0993	0.2388
	Global spontaneity of movement	1.5 ± 0.93	2.25 ± 0.89	2.75 ± 0.71^a^	50.0%	83.3%	0.2841	0.0048	0.6093
**H-Y**		2.38 ± 1.19	2.62 ± 1.06	2.88 ± 0.83	10.1%	21.0%	1	0.9912	1
**Berg**		NA	41.25 ± 9.29	37.38 ± 12.5	\	\			

*Med–, without medication; GPi, globus pallidus interna; STN, the subthalamic nucleus; STN+GPi–, unilateral STN stimulation turning on with contralateral GPi turning off; STN–GPi+, unilateral GPi stimulation turning on with contralateral STN turning off; UPDRS-III, MDS Unified Parkinson Disease Rating Scale part III; H-Y, Hoehn-Yahr stage; Berg, Berg Balance Scare. The formula of percentage of change was (postoperative score–preoperative score)/preoperative score*.

a,b *the letter a indicates a significant difference (P <0.05) between 2 time points (baseline and follow-up), and b indicates a significant difference (P <0.05) between STN+GPi– and STN–GPi+ (paired Student's t-test or Wilcoxon signed-rank test with Bonferroni correction). Values are presented as mean ± SD*.

### Acute Effects of Unilateral STN+/Med+ vs. GPi+/Med+

We then compared the therapeutic effects of STN+/Med+ and GPi+/Med+ after antiparkinsonian medicines were administered. The mean total MDS UPDRS-III score was almost identical to that in the preoperative Med+ condition in the STN+/Med+ state, while there was a worsening in the GPi+/Med+ state. Symptoms dramatically improved on both sides of the body in the STN+/Med+ state, except for the tremor symptoms on the STN-stim side, which showed worsening instead. The improvement of the limbs in the GPi+/Med+ state was more expressive on tremor and rigidity on the GPi-stim side. Similar to that in the Med- state, compared to the preoperative Med+ state, axial symptoms were aggravated in both STN+/Med+ and GPi+/Med+ states (Table 3).

**Table 3:** Motor symptoms in Med+STN+GPi– and Med+STN–GPi+ conditions before and 2 to 3 years after surgery (*n* = 7).

		**Baseline Med+^a^**	**Follow-up Med+**		**Percentage of change**		**Adjusted** ***P*****-value**		
			**STN+GPi–^b^**	**STN–GPi+**	**STN+GPi–**	**STN–GPi+**	**STNa**	**GPi a**	**b**
**Total UPDRS-III**		36.57 ± 21.82	35.29 ± 14.42	43.14 ± 15.49^b^	−3.5%	18.0%	1	0.8655	0.0936
Tremor		4.71 ± 4.11	5.14 ± 3.58	5.43 ± 3.78	9.1%	15.3%	1	1	1
Rigidity		10.29 ± 5.65	7.43 ± 4.58	10.57 ± 3.95^b^	−27.8%	2.7%	1	1	0.1272
Bradykinesia		13.43 ± 11.27	10.29 ± 7.99	14.43 ± 9.83	−23.4%	7.4%	1	1	0.1932
**STN-stim limb**	Tremor	1.57 ± 1.51	2.14 ± 1.35	2.43 ± 1.9	36.3%	54.8%	0.6924	1	1
	Rigidity	4.43 ± 2.15	2.57 ± 1.9	4.43 ± 1.72^b^	−42.0%	0.0%	0.5571	1	0.0321
	Bradykinesia	7.29 ± 5.71	5.14 ± 3.58	8.14 ± 4.6^b^	−29.5%	11.7%	1	1	0.0084
**GPi-stim limb**	Tremor	2 ± 1.29	1.71 ± 1.8	1.57 ± 1.81	−14.5%	−21.5%	1	1	1
	Rigidity	3.86 ± 2.41	2.71 ± 2.21	3.14 ± 1.57	−29.8%	−18.7%	1	1	1
	Bradykinesia	6.14 ± 5.81	5.14 ± 5.15	6.29 ± 5.71	−16.3%	2.4%	1	1	1
**Axial signs**	Total axial score	8.14 ± 5.9	12.43 ± 4.58^a^	12.71 ± 4.42^a^	52.7%	56.1%	0.0954	0.0558	1
	Speech	0.43 ± 0.53	1.14 ± 0.38^a^	1.29 ± 0.49^a^	165.1%	200.0%	0.0699	0.1431	1
	Facial expression	1.57 ± 0.79	2 ± 0.58	2.14 ± 0.38	27.4%	36.3%	1	1	1
	Arising from chair	1 ± 1	0.71 ± 0.49	0.71 ± 0.49	−29.0%	−29.0%	1	1	1
	Gait	1.29 ± 1.25	1.57 ± 0.79	1.43 ± 0.53	21.7%	10.9%	0.8895	1	1
	Freezing of gait	0 ± 0	0.71 ± 0.76^a^	0.43 ± 0.79	∞	∞	0.0888	1	1
	Postural stability	1.14 ± 1.68	2 ± 1.63^a^	2.14 ± 1.21^a^	75.4%	87.7%	0.6939	0.195	1
	Posture	1.86 ± 1.07	2.14 ± 1.21	2.29 ± 0.95	15.1%	23.1%	1	0.4467	1
	Global spontaneity of movement	0.86 ± 1.07	2.14 ± 0.9^a^	2.29 ± 0.95^a^	148.8%	166.3%	0.1179	0.0915	1
**H-Y**		2.43 ± 1.27	2.43 ± 0.98	2.57 ± 0.98	0.0%	5.8%	1	1	1
**Berg**		NA	44.43 ± 7.74	43.14 ± 8.71	\	\			

*Med+, with medication on; GPi, globus pallidus interna; STN, the subthalamic nucleus; STN+GPi–, unilateral STN stimulation turning on with contralateral GPi turning off; STN–GPi+, unilateral GPi stimulation turning on with contralateral STN turning off; UPDRS-III, MDS Unified Parkinson Disease Rating Scale part III; H-Y, Hoehn-Yahr stage; Berg, Berg Balance Scare. The formula of percentage of change was (postoperative score–preoperative score) /preoperative score*.

a,b *the letter a indicates significant difference (P <0.05) between 2 time points (baseline and follow-up), and b indicates a significant difference (P <0.05) between STN+GPi– and STN–GPi+ (paired Student's t-test or Wilcoxon signed-rank test with Bonferroni correction). Values are presented as mean ± SD*.

### Comparison of Unilateral STN vs. GPi DBS on Balance Function (BBS)

We directly compared unilateral STN vs. GPi stimulation on BBS scores in the Med– and Med+ states. In the Med–STN+GPi– condition, the mean score of BBS was 41.25, while it was 37.38 in the Med–STN–GPi+ condition. In the Med+ state, the mean score was 44.43 in the STN+ condition, and 43.14 in the GPi+ conditions (Tables 1, 2).

### Comparison of Patient Groups With Symmetric and Asymmetric Symptoms

In the preoperative Med– and Med+ states, the symmetric group had more severe motor symptoms compared to the asymmetric group. In contrast, in all four postoperative assessment states, the symmetric group showed better improvement in overall motor symptoms for both unilateral STN+ and GPi+ states. In addition, the treatment outcomes on both body sides of the symmetric group outperformed those of the asymmetric group (Supplementary Tables 2, 3).

### Effects of Asymmetric Target DBS on Medication (LEDD)

We also compared medication consumptions before and at the 2- to 3-year follow-up. Compared to the preoperative period, a significant decrease of medication intake was observed at the 2- to 3-year follow-up (26.6%). Six patients had reduced drug use, while two had a slight increase in medication intake (Supplementary Table 4).

### Side Effects

After surgery, some patients experienced transient localized tingling and numbness that got resolved after parameter adjustment. This study focused on the acute effects of unilateral STN and unilateral GPi DBS on motor function in individual PD. When the DBS was turned off either bilaterally or unilaterally, or drug intake was stopped overnight, some patients experienced uncomfortable exacerbations of motor symptoms, including intense tremors, rigidity, and exacerbations of axial symptoms. However, these exacerbations served as observations in this study, which were not recorded as adverse side effects. Moreover, all patients resumed bilateral DBS and medication administration at the end of the trial, and these exacerbations disappeared subsequently. No significant worsening other than motor symptoms was noted after DBS was turned off. No other side effects were observed throughout the study.

## Discussion

In this study, we found that unilateral STN stimulation had a better effect than did unilateral GPi stimulation on improving most cardinal motor symptoms and axial symptoms in both Med– and Med+ states. STN stimulation acted on both sides of the body, whereas GPi stimulation mainly affected the contralateral side. The effects on balance function of STN+ and GPi+ were not significantly different between the Med+ and Med– conditions.

We also found that the improvement in motor symptoms in the Med– state before and after surgery was greater than that in the Med+ state before and after surgery, which was consistent with previous studies (12, 13). Most relevantly, our results suggest that STN is more advantageous than GPi in the treatment in all cardinal symptoms, which conflicts with the previous reports indicating that the two have similar effects (14–19). This may be because we compare the effects of two targets within one patient, reducing bias caused by differences before different cohorts.

Additionally, we found that STN had a effect on both body sides. In contrast, GPi had a treatment effect mainly on the GPi-stim side, while the effects on the STN-stim side were subtle. Previous studies have reported the phenomenon of “dominant STN,” whereby, in some patients, unilateral STN stimulation improved motor symptoms in ipsilateral side, comparable to the effects of bilateral STN stimulation (20–25). However, no similar reportsof dominant GPi have been documented before, although there is a study claiming that the improvement in ipsilateral motor scores from unilateral STN- and GPi-DBS does not differ (26).

Our study also revealed an advantage of STN stimulation in axial symptoms. Previous studies on the therapeutic effects of DBS on axial symptoms have inconsistent results. In general, STN DBS might provide greater alleviation of axial symptoms than GPi DBS; rather, GPi DBS might be associated with a milder long-term decline with regard to these symptoms (3, 27). However, at the 2- to 3-year follow-up, our study indicated less worsening of STN on axial symptoms compared to GPi, which was partly consistent with the findings of previous studies. However, at the same time, the worsening of axial symptoms in both unilateral STN and GPi on conditions may imply that the deterioration mainly come from the disease progression itself. Balance has often been related to postural stability in previous studies (27). In our study, there was little difference in the balance function between STN and GPi stimulation.

Combined unilateral STN and contralateral GPi DBS was originally designed for patients with asymmetric symptoms (5). However, in the subgroup analysis of this study, we found that under unilateral DBS stimulation, patients with symmetric symptoms showed better treatment effects than those with asymmetric symptoms. This may be because the preoperative symptoms in patients in the symmetric group were worse than those in the asymmetric group, leaving more room for improvement. But this may indicate that asymmetric targets can be used equally well in the treatment of patients with symmetric symptoms.

The present results are in line with our previous findings (5) that medication reduction can be achieved by this approach, which may be particularly relevant to target selection for patients who have a pressing need for medication reduction and suffer from contralateral dyskinesia, mood disorders, or worsening cognition.

This study has some limitations. The presence of a biased patient sample and confounding variables cannot be excluded because the study involved a small number of patients. The small sample size implies that the statistical power was sufficient to detect relatively large clinical effects but was insufficient to distinguish between small and subtle effects. Furthermore, we did not conduct studies on STN–GPi– conditions because patients were unable to cooperate with the evaluation due to the sudden worsening of symptoms, which made us obtain the corresponding results indirectly. Nevertheless, this study adopted a new method to compare between different targets within the same patient, namely the “N-of-1” design, which can reduce the interference of PD heterogeneity among different patients. In the future, the synergy of asymmetric targets needs to be assessed in greater depth. Furthermore, the influence of different targets on cognition and neuropsychology can also be researched using the methods described in this article.

## Data Availability Statement

The raw data supporting the conclusions of this article will be made available by the authors, without undue reservation.

## Ethics Statement

The studies involving human participants were reviewed and approved by the Ethical Committee in Ruijin Hospital, Shanghai Jiao Tong University School of Medicine. The patients/participants provided their written informed consent to participate in this study.

## Author Contributions

CZ, DL, and LW designed and conceptualized the study. ZZ, LW, LX, PH, and YP organized and executed the process of the study. WS, ZC, and YL designed and implemented the data analysis. ZZ and KR reviewed the data. ZZ wrote the manuscript. ZL and XX reviewed and performed the language revision. BS, CZ, and DL reviewed and critiqued the manuscript. All authors have approved the final version of the manuscript.

## Funding

DL was sponsored by the National Natural Science Foundation of China (Grant Number 81971294 to DL) and the Shanghai Science and Technology Commission International Cooperation Project (Grant Number 20410712000 to DL). CZ was supported by the fellowship of the Shanghai Research Center for Brain Science and Brain-Inspired Technology and was sponsored by the Natural Science Foundation of China (Grant 82101547 to CZ) and the Shanghai Sailing Program (20YF1426500 to CZ). Gyenno Science Co., LTD., Shenzhen, China, provided database services and data analysis support for this paper and were not involved in the clinical treatment and patient assessment process. The funder was not involved in the study design, collection, analysis, interpretation of data, the writing of this article, or the decision to submit it for publication.

## Conflict of Interest

WS, ZC, YL, and KR were employed by Gyenno Science Co., LTD., Shenzhen. The remaining authors declare that the research was conducted in the absence of any commercial or financial relationships that could be construed as a potential conflict of interest.

## Publisher's Note

All claims expressed in this article are solely those of the authors and do not necessarily represent those of their affiliated organizations, or those of the publisher, the editors and the reviewers. Any product that may be evaluated in this article, or claim that may be made by its manufacturer, is not guaranteed or endorsed by the publisher.

## Abbreviations

DBS: deep brain stimulation
GPi: globus pallidus interna
MDS UPDRS-III: Movement Disorder Society-Unified Parkinson Disease Rating Scale part III
LEDD: levodopa equivalent daily dose
PD: Parkinson's disease
STN: subthalamic nucleus
“+”: med/stimulation on
“−”: med/stimulation off.

## References

[1] Weaver FM Follett K Stern M Hur K Harris C Marks WJ. Bilateral deep brain stimulation vs best medical therapy for patients with advanced Parkinson disease: a randomized controlled trial. JAMA. (2009) 301:63–73. 10.1001/jama.2008.929 19126811 PMC2814800

[2] Obeso JA Olanow CW Rodriguez-Oroz MC Krack P Kumar R Lang AE. Deep-brain stimulation of the subthalamic nucleus or the pars interna of the globus pallidus in Parkinson's disease. N Engl J Med. (2001) 345:956–63. 10.1056/NEJMoa000827 11575287

[3] Ramirez-Zamora A Ostrem JL. Globus pallidus interna or subthalamic nucleus deep brain stimulation for parkinson disease: a review. JAMA Neurol. (2018) 75:367–72. 10.1001/jamaneurol.2017.4321 29356826

[4] Odekerken VJ van Laar T Staal MJ Mosch A Hoffmann CF Nijssen PC. Subthalamic nucleus versus globus pallidus bilateral deep brain stimulation for advanced Parkinson's disease (NSTAPS study): a randomised controlled trial. Lancet Neurol. (2013) 12:37–44. 10.1016/S1474-4422(12)70264-8 23168021

[5] Zhang C Wang L Hu W Wang T Zhao Y Pan Y. Combined unilateral subthalamic nucleus and contralateral globus pallidus interna deep brain stimulation for treatment of parkinson disease: a pilot study of symptom-tailored stimulation. Neurosurgery. (2020) 87:1139–47. 10.1093/neuros/nyaa201 32459849 PMC7666906

[6] Taba HA Wu SS Foote KD Hass CJ Fernandez HH Malaty IA. A closer look at unilateral versus bilateral deep brain stimulation: results of the national institutes of health COMPARE cohort. J Neurosurg. (2010) 113:1224–9. 10.3171/2010.8.JNS10312 20849215

[7] Martínez-Fernández R Máñez-Miró JU Rodríguez-Rojas R Del Álamo M Shah BB Hernández-Fernández F. Randomized trial of focused ultrasound subthalamotomy for parkinson's disease. N Engl J Med. (2020) 383:2501–13. 10.1056/NEJMoa2016311 33369354

[8] Serrien DJ Ivry RB Swinnen SP. Dynamics of hemispheric specialization and integration in the context of motor control. Nat Rev Neurosci. (2006) 7:160–6. 10.1038/nrn1849 16429125

[9] Lizarraga KJ Luca CC De Salles A Gorgulho A Lang AE Fasano A. Asymmetric neuromodulation of motor circuits in Parkinson's disease: the role of subthalamic deep brain stimulation. Surg Neurol Int. (2017) 8:261. 10.4103/sni.sni_292_17 29184712 PMC5680653

[10] Fabbri M Coelho M Guedes LC Rosa MM Abreu D Gonçalves N. Acute response of non-motor symptoms to subthalamic deep brain stimulation in Parkinson's disease. Parkinsonism Relat Disord. (2017) 41:113–7. 10.1016/j.parkreldis.2017.05.003 28528805

[11] Goetz CG Tilley BC Shaftman SR Stebbins GT Fahn S Martinez-Martin P. Movement Disorder Society-sponsored revision of the unified Parkinson's disease rating scale (MDS-UPDRS): scale presentation and clinimetric testing results. Mov Disord. (2008) 23:2129–70. 10.1002/mds.22340 19025984

[12] Williams A Gill S Varma T Jenkinson C Quinn N Mitchell R. Deep brain stimulation plus best medical therapy versus best medical therapy alone for advanced Parkinson's disease (PD SURG trial): a randomised, open-label trial. Lancet Neurol. (2010) 9:581–91. 10.1016/S1474-4422(10)70093-4 20434403 PMC2874872

[13] Schuepbach WM Rau J Knudsen K Volkmann J Krack P Timmermann L. Neurostimulation for Parkinson's disease with early motor complications. N Engl J Med. (2013) 368:610–22. 10.1056/NEJMc1303485 23406026

[14] Okun MS Fernandez HH Wu SS Kirsch-Darrow L Bowers D Bova F. Cognition and mood in Parkinson's disease in subthalamic nucleus versus globus pallidus interna deep brain stimulation: the COMPARE trial. Ann Neurol. (2009) 65:586–95. 10.1002/ana.21596 19288469 PMC2692580

[15] Follett KA Weaver FM Stern M Hur K Harris CL Luo P. Pallidal versus subthalamic deep-brain stimulation for Parkinson's disease. N Eng J Med. (2010) 362:2077–91. 10.1056/NEJMoa0907083 20519680

[16] Zahodne LB Okun MS Foote KD Fernandez HH Rodriguez RL Wu SS. Greater improvement in quality of life following unilateral deep brain stimulation surgery in the globus pallidus as compared to the subthalamic nucleus. J Neurol. (2009) 256:1321–9. 10.1007/s00415-009-5121-7 19363633 PMC3045861

[17] Anderson VC Burchiel KJ Hogarth P Favre J Hammerstad JP. Pallidal vs subthalamic nucleus deep brain stimulation in Parkinson disease. Arch Neurol. (2005) 62:554–60. 10.1001/archneur.62.4.554 15824252

[18] Katz M Luciano MS Carlson K Luo P Marks WJ Larson PS. Differential effects of deep brain stimulation target on motor subtypes in Parkinson's disease. Ann Neurol. (2015) 77:710–9. 10.1002/ana.24374 25627340

[19] Weaver FM Follett KA Stern M Luo P Harris CL Hur K. Randomized trial of deep brain stimulation for Parkinson disease: thirty-six-month outcomes. Neurology. (2012) 79:55–65. 10.1212/WNL.0b013e31825dcdc1 22722632 PMC3385495

[20] Castrioto A Meaney C Hamani C Mazzella F Poon YY Lozano AM. The dominant-STN phenomenon in bilateral STN DBS for Parkinson's disease. Neurobiol Dis. (2011) 41:131–7. 10.1016/j.nbd.2010.08.029 20826212

[21] Agostino R Dinapoli L Modugno N Iezzi E Gregori B Esposito V. Ipsilateral sequential arm movements after unilateral subthalamic deep-brain stimulation in patients with Parkinson's disease. Mov Disord. (2008) 23:1718–24. 10.1002/mds.22203 18661566

[22] Kumar R Lozano AM Sime E Halket E Lang AE. Comparative effects of unilateral and bilateral subthalamic nucleus deep brain stimulation. Neurology. (1999) 53:561–6. 10.1212/WNL.53.3.561 10449121

[23] Linazasoro G Van Blercom N Lasa A. Unilateral subthalamic deep brain stimulation in advanced Parkinson's disease. Mov Disord. (2003) 18:713–6. 10.1002/mds.10407 12784280

[24] Chung SJ Jeon SR Kim SR Sung YH Lee MC. Bilateral effects of unilateral subthalamic nucleus deep brain stimulation in advanced Parkinson's disease. Eur Neurol. (2006) 56:127–32. 10.1159/000095704 16960454

[25] Tabbal SD Ushe M Mink JW Revilla FJ Wernle AR Hong M. Unilateral subthalamic nucleus stimulation has a measurable ipsilateral effect on rigidity and bradykinesia in Parkinson disease. Exp Neurol. (2008) 211:234–42. 10.1016/j.expneurol.2008.01.024 18329019 PMC2413293

[26] Shemisa K Hass CJ Foote KD Okun MS Wu SS Jacobson CEt. Unilateral deep brain stimulation surgery in Parkinson's disease improves ipsilateral symptoms regardless of laterality. Parkinsonism Relat Disord. (2011) 17:745–8. 10.1016/j.parkreldis.2011.07.010 21856205 PMC3791592

[27] Fasano A Aquino CC Krauss JK Honey CR Bloem BR. Axial disability and deep brain stimulation in patients with Parkinson disease. Nat Rev Neurol. (2015) 11:98–110. 10.1038/nrneurol.2014.252 25582445

