# Supplementary material for: Effects of Unilateral Stimulation in Parkinson's Disease: A Randomized Double-Blind Crossover Trial
Source: Front Neurol. 2022 Jan 20;12:812455. doi: 10.3389/fneur.2021.812455 (PMC8812849; doi:10.3389/fneur.2021.812455)
Supplement: Supplementary file 1 [file Data_Sheet_1.PDF]

Supplemental Material 1. Surgical procedure and contact localization

A Leksell stereotactic frame (Elekta, Stockholm, Sweden) was mounted on the patient's head under local anesthesia prior to computed tomography (CT) scanning. The fusion image was obtained by merging the CT and 3.0 T magnetic resonance imaging (MRI) images using Surgiplan software (Elekta, Stockholm, Sweden). Bilateral leads (Medtronic 3387/3389 [Medtronic, Dublin, Ireland] or PINS L302 and PINS 302 [Beijing PINS Medical Co, Beijing, China]) were implanted simultaneously under general anesthesia, and intraoperative C-arm radiography was performed to verify the depth of the electrodes. The system impedance was measured prior to closing the incision. Postoperative brain imaging (CT or MRI) was performed to confirm the lead position and rule out hemorrhage and intracranial pneumatosis.

Supplemental Table 1. Stereotactic coordinates and programming parameters in each patient

Patients	Target	Stereotactic coordinates* (x/y/z) R-lead, L-lead	Programming parameters A/C	Programming parameters A(V)/F(Hz)/P(μs)
Patient 1	R-GPi	78.3/107/105	C+2−3−	2.75/160/90
Patient 1	L-STN	114/102/101	C+8−9−	3.0/160/70
Patient 2	R-STN	86.4/91.5/102	ILS1:C+1−2−; ILS2: C+1−	ILS1: 4.0/115/60; ILS2: 3.0/125/50
Patient 2	L-GPi	119/96.8/99.7	ILS1:C+5−6−; ILS2: 5−6+	ILS1: 3.5/125/30; ILS2: 2.5/125/60
Patient 3	R-GPi	78.6/102/108	C+0−1−	3.15/125/60
Patient 3	L-STN	110/97.4/105	ILS1: C+8−9−; ILS2:C+11−	ILS1:2.6/125/60; ILS2: 2.7/125/70
Patient 4	R-GPi	81.6/100/108	C+1−	3.9/105/90
Patient 4	L-STN	113/94.3/109	ILS1: C+9−10−; ILS2: C+8−	ILS1: 3.5/105/60; ILS2: 2.0/105/60
Patient 5	R-STN	86.3/101/105	C+1−2−	3.35/110/60
Patient 5	L-GPi	120/107/106	ILS1:C+8−9−; ILS2: C+11−	ILS1: 2.85/110/70; ILS2: 3.5/110/90
Patient 6	R-STN	90.6/89.9/107	C+0−1−	3.0/160/60
Patient 6	L-GPi	122/95.9/108	C+8−	3.0/160/60
Patient 7	R-GPi	83.9/105/104	ILS1: C+2−3−; ILS2: C+1−	ILS1: 4.0/125/90; ILS2: 3.0/125/70
Patient 7	L-STN	115/97.2/106	C+8−9−	3.0/125/60
Patient 8	R-GPi	80.7/98.4/103	C+0−2−	3.4/130/70
Patient 8	L-STN	111/91.5/104	C+8−9−	3.45/130/60

R-GPi, right unilateral stimulation of the globus pallidus interna; L-STN, left unilateral stimulation of the subthalamic nucleus; R-STN, right unilateral stimulation of the subthalamic nucleus; L-GPi, left unilateral stimulation of the globus pallidus interna; A/C, anode/cathode; ILS, Interleaving stimulation; A(V)/F(Hz)/P(μs), amplitude(V)/frequency(Hz)/pulse width(μs).

*Stereotactic coordinates are actual postoperative lead locations confirmed by fusion of preoperative and postoperative images in relation to midcommissural point.

Supplemental Table 2. Comparison between patients with asymmetric and symmetric symptoms of changes of motor symptoms in Med−STN+GPi− and Med−STN−GPi+ conditions before and 2 to 3 years after surgery

		Baseline Med−		2−year follow−up Med− STN+GPi−		2−year follow−up Med− STN−GPi+	
		Asymmetric group (*n*=4)	Symmetric group (*n*=4)	Asymmetric group (*n*=4)	Symmetric group (*n*=4)	Asymmetric group (*n*=4)	Symmetric group (*n*=4)
**Total UPDRS-III**		39.5 ± 19.54	68.75 ± 21.72	−6.25 ± 9.95	−21.5 ± 29.46	8.5 ± 10.12	−10.75 ± 24.24
Tremor		7.5 ± 4.04	13.25 ± 9.29	−3.25 ± 0.96	−8 ± 12.96	−2.5 ± 6.45	−4.25 ± 6.9
Rigidity		9.75 ± 4.19	13.75 ± 4.19	−0.5 ± 5.8	−6 ± 5.66	2.5 ± 5.26	−1.75 ± 2.63
Bradykinesia		16.75 ± 8.06	26 ± 7.35	−6.25 ± 5.5	−9 ± 6.68	1.25 ± 5.44	−6.75 ± 8.62
**STN-stim limb**	Tremor	4 ± 1.83	6 ± 3.74	−2.5 ± 0.58	−3.75 ± 5.25	−1 ± 3.46	−2 ± 2.94
	Rigidity	5 ± 2.16	5.25 ± 1.71	−1.5 ± 2.38	−3.75 ± 2.63	0.5 ± 2.08	−0.5 ± 1.91
	Bradykinesia	10.25 ± 3.3	13.25 ± 3.1	−5.75 ± 3.4	−5 ± 1.83	0.75 ± 3.77	−1.5 ± 5
**GPi-stim limb**	Tremor	1.5 ± 1.29	4.25 ± 3.4	0.5 ± 1.29	−2.25 ± 5.12	−0.5 ± 1	−1.25 ± 2.75
	Rigidity	3.5 ± 1.73	5.25 ± 1.26	0 ± 2.45	−2 ± 1.83	0.25 ± 2.5	−1.5 ± 1
	Bradykinesia	6.5 ± 4.8	12.75 ± 4.43	−0.5 ± 2.65	−4 ± 4.9	0.5 ± 3	−5.25 ± 4.43
**Axial signs**	Total axial score	5.5 ± 4.04	15.75 ± 6.02*	3.75 ± 3.2	1.5 ± 6.45	7.25 ± 2.75	2 ± 8.37
	Speech	0.5 ± 0.58	1.75 ± 1.26	0.75 ± 0.5	−0.25 ± 1.5	1.25 ± 0.5	0 ± 1.41
	Facial expression	1.25 ± 0.96	2.5 ± 1	0.75 ± 0.96	−0.25 ± 1.26	1.25 ± 0.5	−1 ± 1.15*
	Arising from chair	0.5 ± 0.58	2 ± 1.83	0 ± 0	−0.75 ± 2.22	0.25 ± 0.5	0 ± 2.58
	Gait	1 ± 0.82	2.25 ± 1.26	0.25 ± 0.5	−0.25 ± 1.26	0.5 ± 0.58	−0.25 ± 1.26
	Freezing of gait	0 ± 0	0 ± 0	0.25 ± 0.5	1 ± 0.82	0.25 ± 0.5	1.25 ± 0.96
	Postural stability	0.25 ± 0.5	2.25 ± 1.5*	0.5 ± 1.29	1.5 ± 1.29	1.25 ± 0.96	1 ± 1.41
	Posture	1.25 ± 0.96	2.75 ± 0.5*	0.25 ± 0.5	0 ± 0	0.75 ± 0.5	0.25 ± 0.5
	Global spontaneity of movement	0.75 ± 0.5	2.25 ± 0.5*	1 ± 1.15	0.5 ± 0.58	1.75 ± 0.5	0.75 ± 0.5
**H-Y**		1.75 ± 0.5	3 ± 1.41	0 ± 0.82	0.5 ± 1	0.5 ± 0.58	0.5 ± 1.73
**Berg**		NA ± NA	NA ± NA	47.5 ± 8.19	35 ± 5.48	45 ± 5.03	29.75 ± 13.57

Med−, without medication; GPi, globus pallidus interna; STN, the subthalamic nucleus; STN+GPi−, unilateral STN stimulation turning on with contralateral GPi turning off; STN−GPi+, unilateral GPi stimulation turning on with contralateral STN turning off ; UPDRS-Ⅲ, MDS Unified Parkinson Disease Rating Scale part Ⅲ; H-Y, Hoehn-Yahr stage.

Asymmetric group, patients with asymmetry index≥0.15 at either Med− or Med+ conditions before surgery ;Symmetric group, patients with asymmetry index<0.15 at both Med− and Med+ conditions before surgery.

This table reflects the difference between patients with symmetrical and asymmetrical symptoms in the improvement or deterioration of motor symptoms in Med−STN+GPi− and Med−STN−GPi+ conditions compared with the Med− condition before surgery. Among them, improvement is shown as negative value, and the larger the absolute value of negative value, the more improvement; aggravation is shown as positive value, the larger the positive value, the more severe the deterioration.

* indicates a significant difference (*P*<0.05) between 2 groups (independent Student's t-test and Wilcoxon rank-sum test).

Values are presented as mean ± SD.

Supplemental Table 3. Comparison between patients with asymmetric and symmetric symptoms of changes of motor symptoms in Med+STN+GPi− and Med+STN−GPi+ conditions before and 2 to 3 years after surgery

		Baseline Med+		2−year follow−up Med+			
				STN+GPi−		STN−GPi+	
		Asymmetric group (*n*=4)	Symmetric group (*n*=3)	Asymmetric group (*n*=4)	Symmetric group (*n*=3)	Asymmetric group (*n*=4)	Symmetric group (*n*=3)
**Total UPDRS-III**		26.5 ± 21.99	50 ± 15.13	7.75 ± 17.52	−13.33 ± 9.02	12 ± 13.54	−0.67 ± 16.01
Tremor		5.5 ± 5.51	3.67 ± 1.53	0.25 ± 0.96	0.67 ± 2.08	0.25 ± 5.56	1.33 ± 5.13
Rigidity		7.25 ± 5.32	14.33 ± 3.21	1.5 ± 7.51	−8.67 ± 2.52	2.75 ± 5.06	−3 ± 4.58
Bradykinesia		9.5 ± 10.08	18.67 ± 12.5	0.5 ± 8.23	−8 ± 10.15	3.25 ± 7.59	−2 ± 12.77
**STN-stim limb**	Tremor	2 ± 1.83	1 ± 1	0.25 ± 0.5	1 ± 1.73	1 ± 2.83	0.67 ± 2.08
	Rigidity	3.75 ± 2.06	5.33 ± 2.31	−0.25 ± 3.3	−4 ± 2	0.75 ± 2.63	−1 ± 2.65
	Bradykinesia	5 ± 5.66	10.33 ± 5.03	−0.25 ± 5.12	−4.67 ± 6.03	2.5 ± 5.57	−1.33 ± 7.09
**GPi-stim limb**	Tremor	2 ± 1.83	2 ± 0	0 ± 0.82	−0.67 ± 0.58	−0.75 ± 1.71	0 ± 2
	Rigidity	2.5 ± 2.38	5.67 ± 0.58	0.75 ± 2.99	−3.67 ± 0.58	0.5 ± 1.29	−2.33 ± 0.58*
	Bradykinesia	4.5 ± 4.65	8.33 ± 7.51	0.75 ± 3.69	−3.33 ± 4.16	0.75 ± 2.22	−0.67 ± 5.69
**Axial signs**	Total axial score	4.25 ± 2.99	13.33 ± 4.51*	5.5 ± 3.87	2.67 ± 4.51	5.75 ± 2.5	3 ± 5.2
	Speech	0.25 ± 0.5	0.67 ± 0.58	0.75 ± 0.5	0.67 ± 0.58	1 ± 0.82	0.67 ± 0.58
	Facial expression	1.25 ± 0.96	2 ± 0	0.75 ± 0.96	0 ± 0	1 ± 1.15	0 ± 0
	Arising from chair	0.5 ± 0.58	1.67 ± 1.15	0 ± 0	−0.67 ± 1.15	0 ± 0	−0.67 ± 1.15
	Gait	0.75 ± 0.5	2 ± 1.73	0.75 ± 0.5	−0.33 ± 0.58	0.5 ± 0.58	−0.33 ± 1.53
	Freezing of gait	0 ± 0	0 ± 0	0.5 ± 0.58	1 ± 1	0 ± 0	1 ± 1
	Postural stability	0 ± 0	2.67 ± 1.53*	0.75 ± 0.5	1 ± 1	1.25 ± 0.5	0.67 ± 1.53
	Posture	1.25 ± 0.96	2.67 ± 0.58	0.25 ± 0.96	0.33 ± 0.58	0.5 ± 0.58	0.33 ± 0.58
	Global spontaneity of movement	0.25 ± 0.5	1.67 ± 1.15	1.75 ± 0.5	0.67 ± 1.15	1.5 ± 0.58	1.33 ± 1.15
**H-Y**		1.75 ± 0.5	3.33 ± 1.53	0 ± 0.82	0 ± 1	0.25 ± 0.96	0 ± 1.73
**Berg**		NA ± NA	NA ± NA	49.75 ± 4.57	37.33 ± 4.04	46.75 ± 6.02	38.33 ± 10.6*

Med−, without medication; GPi, globus pallidus interna; STN, the subthalamic nucleus; STN+GPi−, unilateral STN stimulation turning on with contralateral GPi turning off; STN−GPi+, unilateral GPi stimulation turning on with contralateral STN turning off ; UPDRS-Ⅲ, MDS Unified Parkinson Disease Rating Scale part Ⅲ; H-Y, Hoehn-Yahr stage.

Asymmetric group, patients with asymmetry index ≥0.15 at either Med− or Med+ conditions before surgery ;Symmetric group, patients with asymmetry index<0.15 at both Med− and Med+ conditions before surgery.

This table reflects the difference between patients with symmetrical and asymmetrical symptoms in the improvement or deterioration of motor symptoms in Med−STN+GPi− and Med−STN−GPi+ conditions compared with the Med+ condition before surgery. Among them, improvement is shown as negative value, and the larger the absolute value of negative value, the more improvement; aggravation is shown as positive value, the larger the positive value, the more severe the deterioration.

* indicates a significant difference (*P*<0.05) between 2 groups (independent Student's t-test and Wilcoxon rank-sum test).

Values are presented as mean ± SD.

Supplemental Table 4. Changes in preoperative and postoperative drug administration (LEDD) in each patient

Patients	LEDD (mg) preoperative	LEDD (mg) postoperative	Changes of LEDD (mg)	Percentage of change
Patient 1	700	673.75	−26.25	-3.75%
Patient 2	525	449	−76	-14.48%
Patient 3	500	250	−250	-50.00%
Patient 4	150	0	−150	-100.00%
Patient 5	425	475	50	11.76%
Patient 6	787.5	798	10.5	1.33%
Patient 7	1050	625	−425	-40.48%
Patient 8	798.25	350	−448.25	-56.15%

LEDD, levodopa equivalent daily dose. Mean preoperative dose of medication, 617.0 ± 276.0 mg; Mean postoperative dose of medication, 452.6 ± 254.9 mg (*P*=0.046, paired Student's t-test)
