# Supplementary material for: Effects of Unilateral Stimulation in Parkinson's Disease: A Randomized Double-Blind Crossover Trial
Source: Front Neurol. 2022 Jan 20;12:812455. doi: 10.3389/fneur.2021.812455 (PMC8812849; doi:10.3389/fneur.2021.812455)
Supplement: Supplementary file 2 [file Data_Sheet_2.docx]

# Protocol and Statistical Analysis Plan

This trial protocol has been provided by the authors to give readers additional information about their work.

Protocol for: Zhitong Zeng, Linbin Wang, Weikun, Shi, et al. Effects of unilateral stimulation in Parkinson’s disease: a randomized double-blind crossover trial.

**This supplement contains the following items:**

1. Original study protocol and statistical analysis plan: page 2-5
2. Final study protocol and statistical analysis plan: page 6-9
3. Changes and reasons: page 10

## 1. Original study protocol and statistical analysis plan

### 1.1 Trial design

This is a prospective, double blind, randomized, self-control, cohort study, designed to compare the differences between unilateral STN stimulation and unilateral GPi stimulation in the same PD patient with symmetric motor symptoms (a left-extremity to right-extremity ratio of less than 0.15 on the UPDRS-Ⅲ in the both off-medication and on-medication states). Participants with advanced PD who had previously undergone combined unilateral STN and contralateral GPi DBS will be screened based on inclusion and exclusion criteria. After recruitment, participants will be comprehensively evaluated under different conditions (acute unilateral GPi turning-on versus acute unilateral STN turning-on in the both off-medication and on-medication states). All participants and trained assessors are blind to the conditions, which are randomly assigned in two continuous days. The study will be carried out under the supervision of the ethical committee of Shanghai Jiao Tong University Ruijin Hospital, and informed consent will be obtained from all participants (see Figure 1).


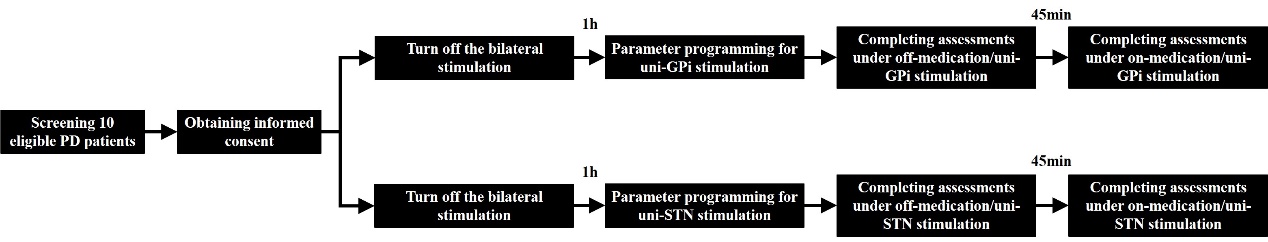


Figure 1 Trial design

### 1.2 Trial population

Participants are recruited from the Departments of Functional Neurosurgery at Ruijin Hospital, Shanghai Jiao Tong University School of Medicine (Shanghai, China).

### 1.3 Patient population and recruitment

We intend to enroll a total of 10 patients. The study will comprise PD patients who have received combined unilateral STN and contralateral GPi DBS.

#### 1.3.1 Inclusion criteria

1. Patients with idiopathic Parkinson's disease
2. Aged between 55 and 75 years, both male and female
3. Patients who have undergone combined unilateral STN and contralateral GPi DBS for more than 2 years
4. A Hoehn-Yahr (H-Y) stage of less than 4 in the off-medication state
5. A left-extremity to right-extremity ratio of less than 0.15 on the Unified Parkinson’s Disease Rating Scale part Ⅲ (UPDRS-Ⅲ) in the both off-medication and on-medication states.

#### 1.3.2 Exclusion criteria

1. History of serious psychosis
2. History of intractable epilepsy (i.e., seizures)
3. Diagnosed by the investigators that patients with severe cardiac, liver and kidney diseases, or other serious health conditions
4. Dementia (A Mini-Mental State Examination (MMSE) score of < 24), inability to comprehend the experimental protocol or voluntarily provide informed consent
5. Lack of cooperation
6. Additional reasons for exclusion at the discretion of the clinical investigator
7. Poorly controlled depression or anxiety
8. Past history of suicidal attempt

#### 1.3.3 Recruitment of participants

Participants will be recruited by phone-call and directly at the department. A well-trained investigator will be responsible for screening all potentially eligible patients based on the eligibility criteria and obtaining the informed consent.

### 1.4 Interventions

#### 1.4.1 Unilateral DBS of the subthalamic nucleus (STN)

To deliver unilateral STN DBS, we will turn off the bilateral stimulation for an hour, and then turn on the unilateral STN DBS. The STN stimulation will be programmed as previous parameter configuration with optimal therapeutic benefits. Participants will be asked to complete a comprehensive set of assessments under unilateral STN stimulation in the off-medication state. One hour after taking regular medication, participants need to complete the second set of assessments in the on-medication state.

#### 1.4.2 Unilateral DBS of the globus pallidus interna (GPi)

To deliver unilateral GPi DBS, we will turn off the bilateral stimulation for an hour, and then turn on the unilateral GPi DBS. The study protocol is identical to the intervention of unilateral STN DBS but it was done on a different day.

#### 1.4.3 Concomitant interventions

Participants will be asked to withhold antiparkinsonian drugs for 12 hours overnight and stay in the off-medication state until they complete the first set of assessments. Participants will be asked to take regular medication for the second set of assessments in the on-medication state. All process will be repeated for contralateral target on the other day. Usage of all drugs will be documented in the Case Report Form (CRF).

### 1.5 Randomization and blinding

Throughout the study, all participants, independent raters/evaluators and statisticians will be blind to the treatment conditions and parameter configurations. Independent raters who are blind to the conditions will be responsible for the conduct of all the assessments. Participants are blind to the treatment conditions. Movement disorders clinician responsible for programming are blind to the study protocol. They are simply told to adjust parameter to optimize therapeutic effects.

Testing sequence of treatment conditions will be randomly assigned in a one-to-one ratio to the scheduled days. Moreover, participants’ hemispheric targets of DBS should be counterbalanced in the left and right side, with half participants having left-STN DBS and half having left-GPi DBS.

### 1.6 Trial outcomes

1. Differences in motor symptoms between two treatment conditions in the on- and off- medication states, as defined by total UPDRS part Ⅲ scores which range from 0 to 132, with higher score indicating more severe motor symptoms.
2. Differences in balance function between two treatment conditions in the on- and off- medication states, as defined by BBS (Burg Balance Scale) scores which range from 0 to 64, with higher score indicating better balance ability.

### 1.7 Data quality and management

Data collection will be restricted to the those meet the eligibility criteria. Participants who withdraw from the study for any reason will be recorded in their medical records and excluded from the data analysis.

#### 1.7.1 Withdrawal and terminate from the study

Participants will be instantly withdrawn from the study in the following conditions:

1. Endangerment of personal safety;
2. Patient lack of compliance;
3. Withdrawal of informed consent;

#### 1.7.2 Handling of missing data

Mean imputation will be used to handle the missing data in the questionnaire. Listwise deletion will be used to handle the missing data that more than 20%. Participant’s specific missing data for one outcome measure will be deleted while his other data can be included in the data analysis, only if the sample size are described in detail.

### 1.8 Statistical considerations

#### 1.8.1 Sample size

The total of 10 patients are expected to be included in this **exploratory** study.

#### 1.8.2 Data analysis

The (nonparametric) Wilcoxon matched-pairs signed-rank test will be used to make pairwise comparisons between treatment conditions. Categorical data will be analyzed with χ2 tests. A p-value < 0.05 will be considered statistically significant.

## 2. Final study protocol and statistical analysis plan

### 2.1 Trial design

This was a prospective double-blind randomized crossover study designed to compare the acute effect of unilateral STN and GPi stimulation on motor symptoms in several patients with PD. Participants with advanced PD who had previously undergone combined unilateral STN and contralateral GPi DBS were screened based on the inclusion and exclusion criteria. Following recruitment, participants were comprehensively evaluated under four randomized, double-blind conditions: (1) Med−STN+GPi−, (2) Med−STN−GPi+, (3) Med+STN+GPi−, and (4) Med+STN−GPi+. The symbol + means on, while − means off. The **intervention** section explains the details of these conditions. All participants and trained assessors were blinded to the conditions, and patients were randomly assessed over the course of two continuous days. (**Figure. 1**)


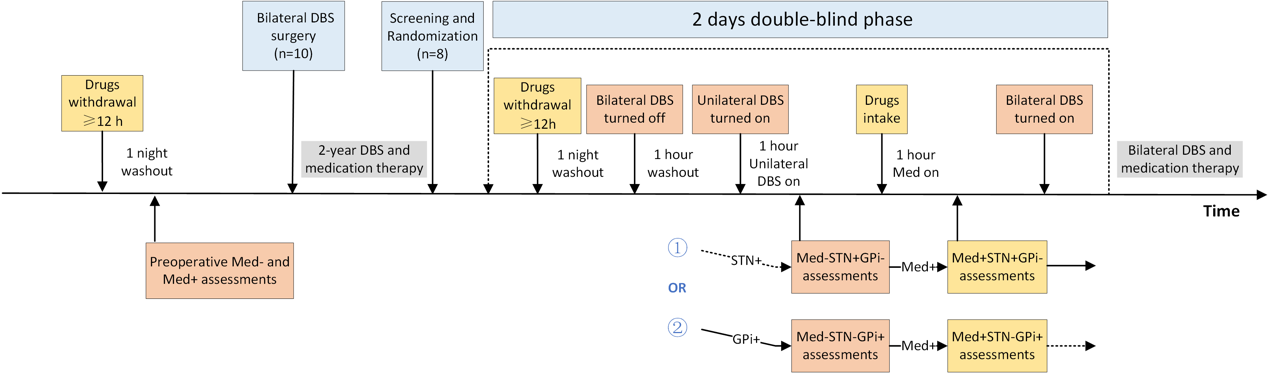


Figure. 1 Trial design.

### 2.2 Patients

Participants were recruited from the Department of Functional Neurosurgery at Ruijin Hospital, Shanghai Jiao Tong University School of Medicine (Shanghai, China). A total of 10 patients with PD underwent combined unilateral STN and contralateral GPi DBS from September 2017 to September 2018. Following recruitment in April 2020 and screening, eight patients who had received the surgery for 2 to 3 years were included in this study. The inclusion criteria were: 1) diagnosis of idiopathic PD; 2) age between 55 and 75 years, both male and female; 3) treatment with combined unilateral STN and contralateral GPi DBS for 2 to 3 years and with optimal parameters for 3 months; and 4) a Hoehn-Yahr (H-Y) stage of less than 4 in the medication-off state. The exclusion criteria were: 1) history of serious psychosis; 2) history of intractable epilepsy (i.e., seizures); 3) diagnosis of severe cardiac, liver or kidney diseases, or other serious health conditions; 4) dementia (A Mini-Mental State Examination score of <24), inability to comprehend the experimental protocol or voluntarily provide informed consent; 5) lack of cooperation; 6) poorly controlled depression or anxiety. The patients in this study overlapped partly with those in our previous study published in 2020;^5^ those were, patients, 3, 7 and 8. Additionally, according to the asymmetry index, patients were divided into a symmetric group (asymmetry index <0.15, both Med− and Med+ conditions before surgery) and an asymmetric group (asymmetry index ≥0.15, either Med− or Med+ conditions before surgery), and the corresponding subgroup analysis was performed. The asymmetry index was a left-extremity to right-extremity ratio in the MDS UPDRS-III, which was calculated using the formula (left extremity – right extremity) / (left extremity + right extremity). A team of experienced multidisciplinary DBS specialists made the clinical decision regarding the specific DBS target to be used in each patient. That was, unilateral STN DBS was applied to treat the more severe side since we hypothesized that STN is more effective than GPi.

### 2.3 Interventions

#### 2.3.1 Unilateral DBS of STN

Bilateral stimulation was turned off for an hour^10^, and unilateral STN DBS was turned on afterwards. Participants were asked to complete a comprehensive set of assessments under unilateral STN stimulation in the Med− state. Participants were further required to complete the second set of assessments in the Med+ state one hour after taking regular medications.

#### 2.3.2 Unilateral DBS of GPi

Unilateral GPi DBS was delivered after bilateral stimulation was turned off for an hour. The study protocol was identical to that used in the unilateral STN DBS intervention but was performed on a different day. After all these assessments, bilateral DBS will be turned on again and returned to normal treatment status.

#### 2.3.3 Concomitant interventions

Participants were asked to stop taking antiparkinsonian drugs for 12 h to stay in the Med− state until they completed the first set of assessments. Regular medication was taken one hour before the second set of assessments to maintain a Med+ state. All processes were repeated for the contralateral target on the next day.

### 2.4 Randomization and blinding

The testing sequence of the treatment conditions was randomly assigned in a counterbalanced manner on the scheduled days. The order of the DBS conditions was determined by the clinician who randomly picked up one of the eight folded sheets with different conditions written on them (half of the first day GPi; half of the first day STN) but was not allowed to participate in any rating or evaluation. Throughout the study, all participants, raters, and statisticians were blinded to treatment conditions. A movement disorder specialist was responsible for programming. In addition, motor symptom evaluation in this experiment was performed by an experimenter who was blinded to the study protocol and did not participate in data analysis or interpretation. Two raters who were blinded to the conditions conducted the video assessments independently, after which the average rating scores were calculated. For subscores with large deviancy, the final scores were determined after re-evaluation.

### 2.5 Trial outcomes

Acute turning-on effects of unilateral STN stimulation versus unilateral GPi stimulation on motor symptoms in each patient were compared as the primary outcome. Motor symptoms were defined by the MDS UPDRS-III scores which ranged from 0 to 132, with higher scores indicating more severe motor symptoms. To gain insight into the specific effects of each target, we classified the MDS UPDRS-III subscales into three categories: 1) axial signs, as measured by scores on speech, facial expression, arising from a chair, posture, gait, freezing of gait, and posture stability; scores could range from 0 (no axial signs) to 28 (severe axial signs); 2) STN-stimulated contralateral limb symptoms; and 3) GPi-stimulated contralateral limb symptoms. Limb symptom severity was measured using the subscale scores of the corresponding limb on rigidity, finger tapping, hand movements, hand pronation supination, toe-tapping, leg agility, posture tremor, kinetic tremor, and resting tremor amplitude; scores could range from 0 (no limb symptoms) to 52 (severe limb symptoms). The Berg Balance Scale (BBS) was also compared as a second outcome at the 2- to 3-year follow-up. The patient’s daily dose of antiparkinsonian medication was converted into a levodopa equivalent daily dose (LEDD).

### 2.6 Data analysis

There were two types of comparisons conducted in this study. The first was the comparison between unilateral STN+ and GPi+ within the same patient group in Med− and Med+ conditions, and the second was the comparison between asymmetric and symmetric groups for the different patient groups in the same condition. Before the comparisons, the Shapiro-Wilk test was used to test the normality of data in each group, yielding the W statistic and *P*-value reflecting the evaluation criteria of distribution. For normally distributed data, a parametric test of the Student’s t-test was used to assess the difference between groups. For the non-normally distributed data, the non-parametric Wilcoxon test was applied to compare the differences. The first comparison mentioned was based on the paired Student’s t-test and Wilcoxon signed-rank test. The second comparison was based on the independent Student’s t-test and Wilcoxon rank-sum test. All three tests mentioned were two-tailed tests with a *P*-value*<0.05* reflective of statistical significance. Statistical calculations and techniques were performed using R-4.0.2.

## 3. Summary of changes and reasons

| **Original study protocol and statistical analysis plan** | **Changes** | **Reasons** |
| --- | --- | --- |
| We originally intended to enroll a total of 10 patients with a left-extremity to right-extremity ratio of less than 0.15 on the MDS UPDRS-III in the both Med on and Med off states. | We modified the previous requirement of an asymmetry index less than 0.15 in the inclusion criteria.  8 patients were eventually enrolled, only half of whom met the asymmetry index of less than 0.15. | DBS treatment with asymmetric targets was firstly used mainly in patients with asymmetric motor symptoms in both limbs in the pilot study. A total of 10 patients met the requirement of a 2- to 3-year follow-up period, but only 8 patients could be included based on the inclusion criteria and patient preference. |
| We originally intended to perform stimulation parameter adjustments prior to assessments. | In practice, we kept the optimal parameters and did not adjust them before the assessment. | Retaining the stable optimal parameters allows a better comparison of the two targets in daily treatment. |
| We originally intended to perform the assessment in the Med on condition 45 minutes after taking the parkinsonism drugs. | In practice, the assessment in Med on condition was performed 1 hour after taking the medication. | Previous evidence suggests that 1 hour may be more reasonable and that the patient is in a better state. |
| The Wilcoxon matched-pairs signed-rank test and χ2 test were considered to be used. | the Shapiro-Wilk test was used to test the normality of data in each group, yielding the W statistic and *P*-value reflecting the evaluation criteria of distribution. For normally distributed data, a parametric test of the Student’s t-test was used to assess the difference between groups. For the non-normally distributed data, the non-parametric Wilcoxon test was applied to compare the differences. | The final statistical analysis is more standardized. |
